# A Computed Tomography Study on the Prevalence of Lusorian Artery Among Hungarian Adults

**DOI:** 10.7759/cureus.58622

**Published:** 2024-04-20

**Authors:** Julia Shamsodini, Dávid Molnár

**Affiliations:** 1 Department of Otolaryngology, Semmelweis University, Budapest, HUN; 2 Department of Anatomy, Histology and Embryology, Semmelweis University, Budapest, HUN; 3 Department of Otorhinolaryngology and Head and Neck Surgery, Central Hospital of Northern Pest - Military Hospital, Budapest, HUN

**Keywords:** lusorian artery, body patterning, thyroidectomy, non-recurrent laryngeal nerve, aberrant right subclavian artery, three-dimensional imaging, computed tomography

## Abstract

Introduction

The aberrant right subclavian artery (ARSA), also called as lusorian artery (LA) is a developmental anomaly that exists in conjunction with a right non-recurrent laryngeal nerve (NRLN) in almost all cases. The average prevalence of such a vascular variation is estimated as 1%, although, studies have reported very different population means. Up to date, there is no available data on the frequency of this pattern in the Hungarian population. It can be treated as an indirect marker of a NRLN. Any preoperative information on the course of the inferior laryngeal nerves can help surgeons reduce the risk of an iatrogenic injury during thyroidectomies, especially in an environment where access to intraoperative neuromonitoring is limited.

Objectives

The primary aims were to determine the prevalence of an ARSA, predict the existence of an NRLN in the Hungarian population, and provide demographic analysis.

Methods

A retrospective, computed tomography-based study was carried out. Demographic description and statistical analysis were provided where applicable. Detected anomalous vasculatures were visualized with 3D segmentation, and images were interpreted.

Results

The imaging database review identified three patients with ARSA out of 686 eligible recordings, resulting in a frequency of 0.437% in the study population. All three patients were female and had a retroesophageal LA. Two of them had a Kommerell's diverticulum. One patient had common carotid arteries with a single origin.

Conclusions

The frequency of an ARSA and a concomitant NRLN among Hungarians fits into the results of recent meta-analyses. Preoperative assessment of this anomaly may reduce vocal cord complication rates of thyroidectomies.

## Introduction

According to the International Recurrent Laryngeal Nerve Classification System, variations of the recurrent laryngeal nerve can be either acquired or embryologic [1]. The most common developmental variation is the non-recurrent laryngeal nerve (NRLN). This symptomless anomaly can occur on the right side where it emerges from the vagus nerve on the neck with a sloping trajectory toward its entry point into the larynx. Thus, the routine surgical approach and the conventional landmarks can be misleading during thyroid surgery.

NRLNs are a type of nerve that occurs when the aortic arch forms abnormally during embryonic development. A right-sided NRLN is believed to be caused by the improper absorption of the fourth right branchial arch. This also results in an aberrant right subclavian artery (ARSA), also known as the lusorian artery (LA) with a left-sided aortic arch. The ARSA does not originate from the brachiocephalic trunk as it does conventionally; instead, it emerges as the last branch of a left-sided aortic arch and passes either between or behind the trachea and esophagus.

Abnormal origin of the right subclavian artery occurs due to the regression of the right fourth branchial vascular arch and the proximal part of the right dorsal aorta in combination with the presence of the seventh intersegmental artery, which arises from the proximal descending thoracic aorta. This leads to the anomalous course of the LA [2]. During embryonic development, the right recurrent laryngeal nerve gets caught under the fourth aortic arch artery, which forms part of the right subclavian artery, since the fifth and sixth arch arteries disappear [3]. In the case of ARSA, there is no fourth arch artery that makes the inferior laryngeal nerve loop around it, thus it emerges in a descending, non-recurrent fashion.

NRLN and ARSA exist only in conjunction; thus, they are parts of a compound phenotype that results from the same, altered developmental process. Nevertheless, NRLN can almost exclusively occur on the right side. Exceptionally, NRLN can develop on the left side in the case of situs inversus totalis with an aberrant left subclavian artery, which is an extremely rare constellation [4]. The underlying mechanism can be translated as a mirror image of a right-sided anomaly. A right-sided aorta can also exist without situs inversus. This variation is almost always associated with an aberrant left subclavian artery. This phenotype is the result of a different underlying chain of embryonic events and, due to its different nature, is not always in combination with a left-sided NRLN [5,6]. Fellmer, based on the observations of Sander, concluded that the combination of a right-sided aortic arch, an aberrant left subclavian artery (ALSA), and the lack of a ductus arteriosus (DA) is essential for a left NRLN [5,7].

Due to the accompanying presence of ARSA and NRLN, prevalence analysis of this vascular anomaly can also describe the frequency of an NRLN in a study population. To our knowledge, there is no available data on the recent prevalence of ARSA in the Hungarian population.

## Materials and methods

Ethics approval

The entire research process was according to the World Medical Association’s Declaration of Helsinki. This study was approved by the Institutional Review Board of the Medical Centre Hungarian Defense Forces (MH EK) and its current legal successor, the Central Hospital of Northern Pest - Military Hospital (ÉPC-HK) (approval no. DA20211201). For convenience’s sake, we uniformly refer to the Central Hospital of Northern Pest - Military Hospital (ÉPC-HK) as the site of this study.

Study design

Study Population

**Figure 1:**
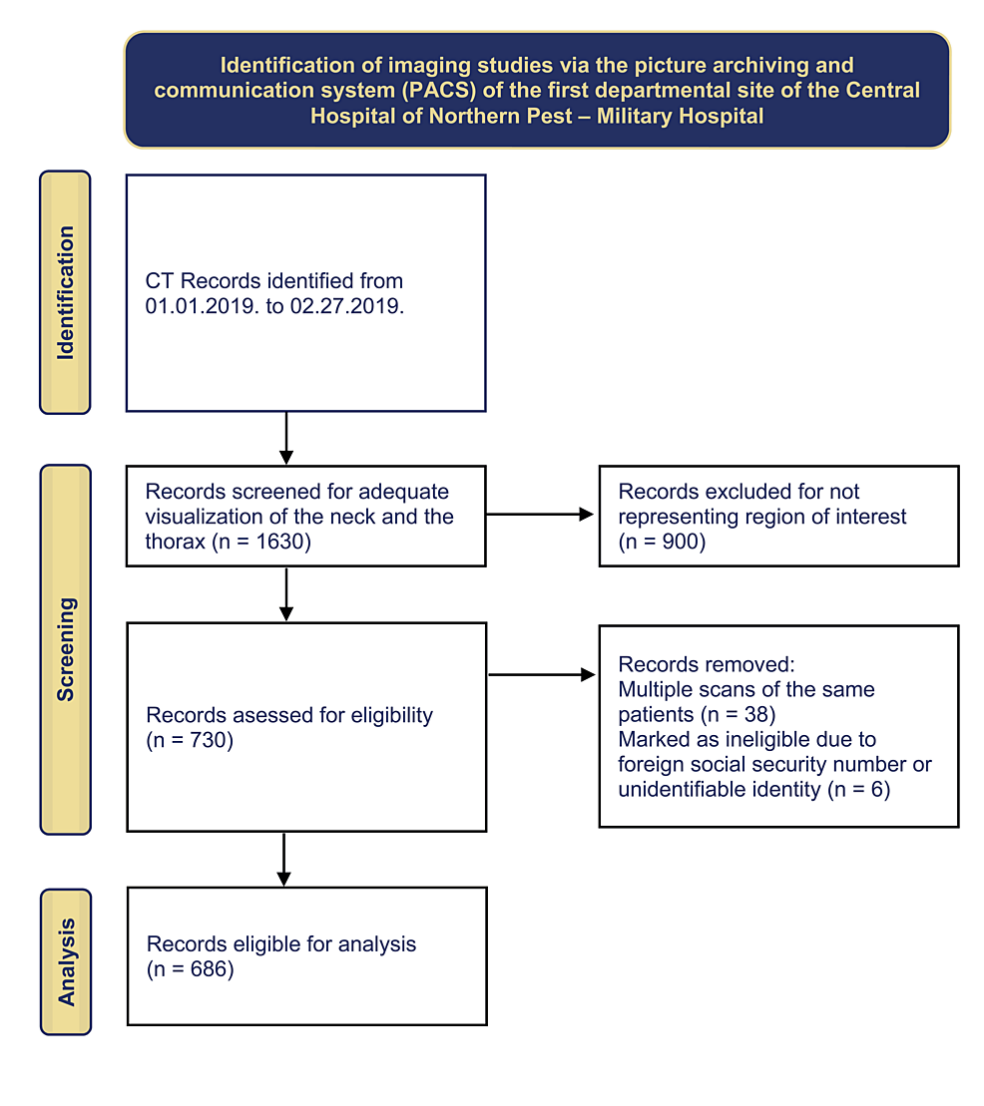
Flowchart of imaging data retrieval and selection

We aimed to retrospectively screen the computed tomography images acquired at the first departmental site of ÉPC-HK from January 1, 2019. The research workflow is visualized (Figure 1).

The hospital’s picture archiving communication system (PACS) was accessed with the JiveX Diagnostic Client v4.6.3 software (VISUS Health IT GmbH, Bochum, Germany). All records were evaluated by either the first author and confirmed by the senior author, or the senior author alone. The review covers the January 1, 2019-February 27, 2019 interval.

About 1630 CT studies were archived in the designated interval. We manually reviewed all cases, regardless of the name of the study protocol whether they adequately cover the lower neck and the superior mediastinum. This is due to the fact that the labels of the study protocols on the main screen table didn't always match the actual spatial extensions of the tomograms; therefore, the aim of this approach was to yield as many feasible scans as possible. Consequently, 900 records were excluded for not representing the region of interest (ROI).

About 730 records were assessed for eligibility. Our focus group was the Hungarian population. Therefore, six studies were marked as ineligible due to foreign social security numbers or unidentifiable identities. Some patients underwent multiple CT scans in the same period. For this reason, another 38 records were also removed as duplicates. About 686 individuals’ data were suitable for analysis (Figure 1).

Image Analysis

The 686 eligible records included enough sections through the lower neck, the thoracic aperture, and the superior mediastinum. Acquisitions were either native or contrast-enhanced CTs with various protocols, e.g., standard chest tomography, pulmonary angiography, carotid angiography, neck soft tissue tomography, polytrauma protocol, and cardio-CT.

Multiplanar reconstructions were prepared on-site with the JiveX Diagnostic Client v4.6.3 software. At-home analysis was done with the RadiAnt DICOM Viewer v2022.1 software (Medixant, Poznan, Poland). Three-dimensional visualizations and segmentations were generated with Slicer 3D v5.0.3 [8].

Images were compiled and edited with Illustrator CS5 (Adobe, San Jose, United States) and Photoshop CS5 (Adobe, San Jose, United States).

Data Processing

Data logs were conducted in Excel 2016 (Microsoft, Redmond, United States) sheets. Data was processed, and descriptive statistics were done by using Excel 2016 and IBM SPSS Statistics for Windows, Version 28 (Released 2021; IBM Corp., Armonk, New York, United States).

## Results

About 686 individuals’ CT scans contained appropriate data for analysis. This study sample showed a 1.054:1 male:female ratio (n_male_ = 352, n_female_ = 334). Age distribution was 63.01 ± 15.194 SD in the male and 64.93 ± 17.493 SD in the female group (Table 1).

**Table 1:** Demographic data of the study population

Sex	Number (n)	Age characteristics (years)			
		Minimum	Maximum	Mean	Standard deviation (SD)
Male	352	18	96	63.01	15.194
Female	334	18	99	64.93	17.493

Three subjects (0.437%) were identified with an ARSA, the indirect sign of having a NRLN on the right side. This prevalence data and the female-only occurrence were not appropriate for statistical analysis (Pearson’s chi-square and Fisher’s exact test; data is submitted in the Appendix). It should be noted, that none of the radiology reports contained a description of LAs.

Table 2 demonstrates the demographic data of the three patients. Subtypes of LA exist including retroesophageal (RE) and retrotracheal (RT) courses. All of the three subjects had a RE route of LA. Incidentalomas are also listed in Table 2.

**Table 2:** Patients’ characteristics identified with aberrant right subclavian artery ID: Subject identifier; M: Male; F: Female; TH: Chest CT; CA: Carotid + Willis CT angiography; RE: Retroesophageal course; RT: Retrotracheal

ID	Gender (M/F)	Age (years)	Scanning modality	Course of lusorian artery (RE/RT)	Incidental findings
36	F	73	TH	RE	aorta aneurysm, Kommerell divertiulum
230	F	69	CA	RE	goiter, common origin of carotid arteries, Kommerell diverticulum
587	F	60	TH	RE	retrosternal goiter

**Figure 2:**
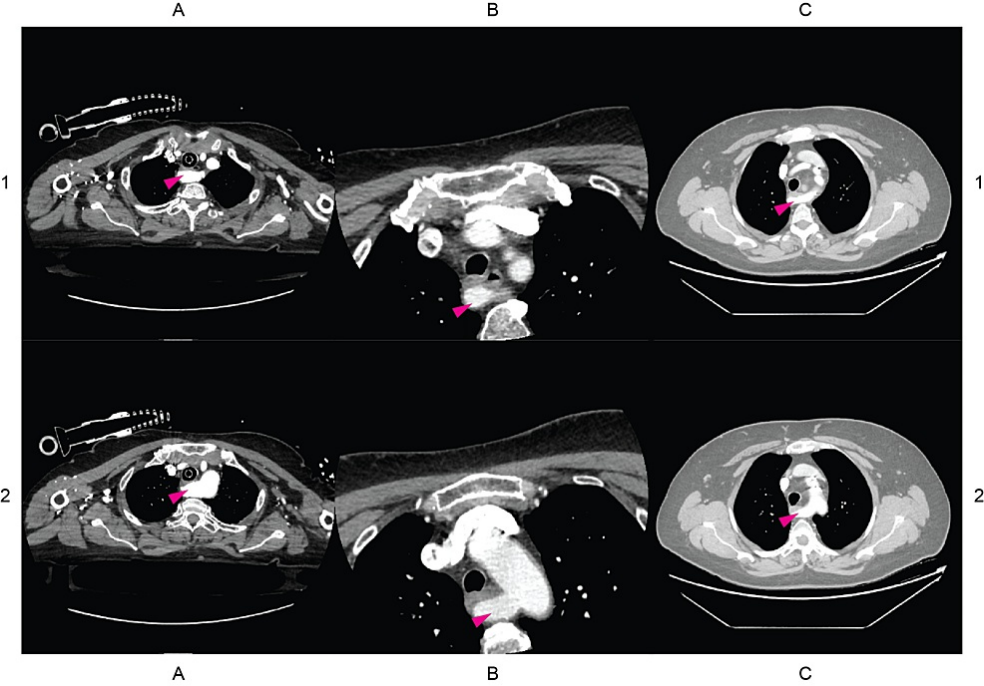
Axial CT images of patients with identifiers ID036, ID230, and ID587 Columns A, B, and C contain raw axial CT images of patients with identifiers ID036, ID230, and ID587 respectively. The crosshairs on the left are for orienting the corresponding row. The magenta arrowheads label the aberrant right subclavian arteries, also called lusorian arteries. Each patient has a retroesophageally traversing lusorian artery resulting in an anterior-posterior compression of the esophagus. Nota Bene (NB): in the C1 image the lower pole of the left thyroid lobe spreads into the thorax approaching the trachea, the esophagus, and the lusorian artery.

**Figure 3:**
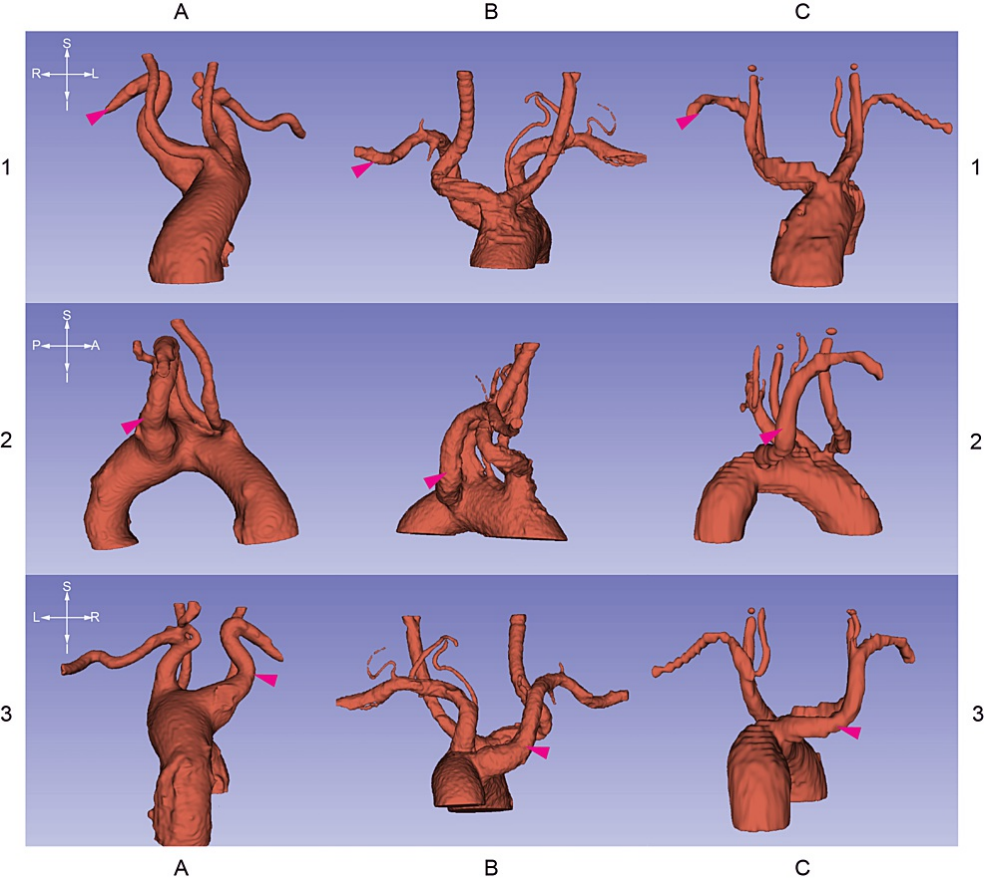
3D segmentations generated from CT stacks Columns A, B, and C are 3D reconstructions of the aortic arch and its major branches of patients with identifiers ID036, ID230, and ID587 respectively. The crosshairs on the left are for orienting the corresponding row. Magenta arrowheads label the aberrant right subclavian arteries. Brachiocephalic trunks are missing. NB: ID036 and ID230 patients have Kommerell diverticulum as visible in 3A and 3B insets, respectively. As B1 and B2 images depict, the common carotid arteries of subject ID230 emerge with a common trunk from the aortic arch.

**Figure 4:**
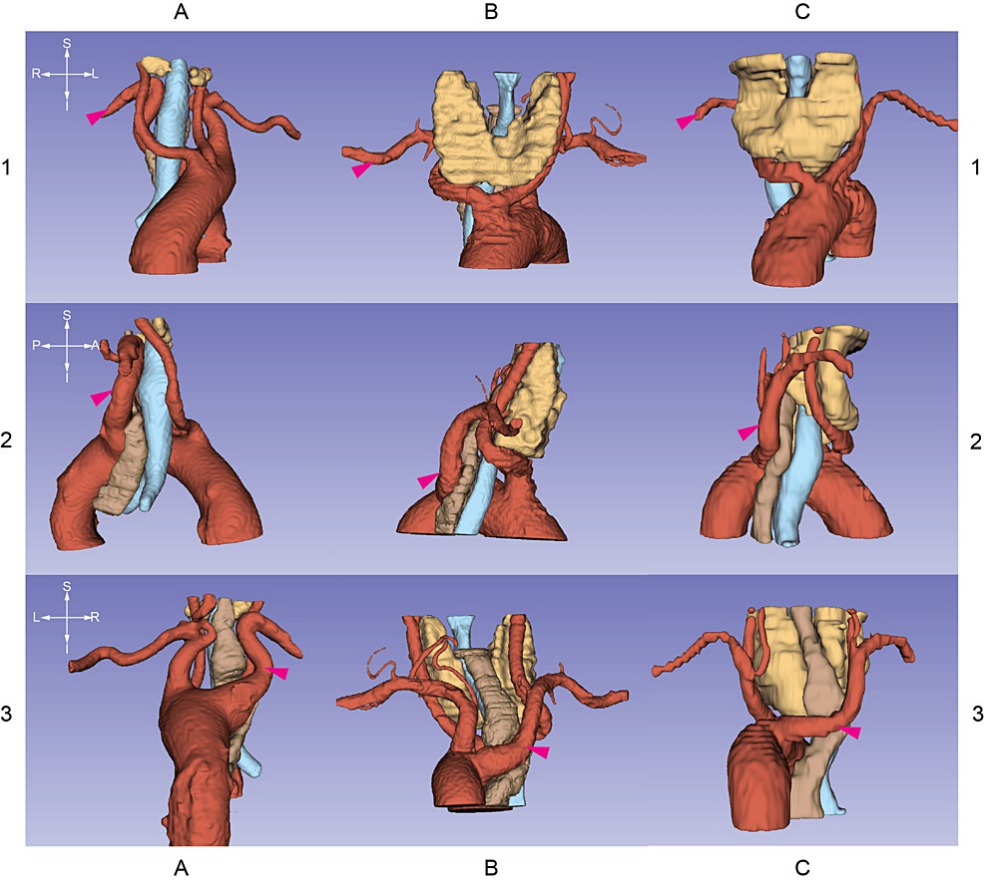
3D segmentations generated from CT stacks Columns A, B, and C are 3D reconstructions generated from CT images of patients with identifiers ID036, ID230, and ID587 respectively. The crosshairs on the left are for orienting the corresponding row. Magenta arrowheads label the aberrant right subclavian arteries. This matrix of images demonstrates the syntopy of the lusorian artery and the surrounding organs including the trachea (light blue), esophagus (brown), and thyroid glands (yellow). All of the detected lusorian arteries traverse behind the esophagus. NB: Patients ID230 and ID587 have goiters expanding the space between the carotid vessels. C1, C2, and C3 show signs of a more prominent thoracic spread of the left lobe of the thyroid gland resulting in a minor tracheal dislocation but not compression in subject ID587.

**Figure 5:**
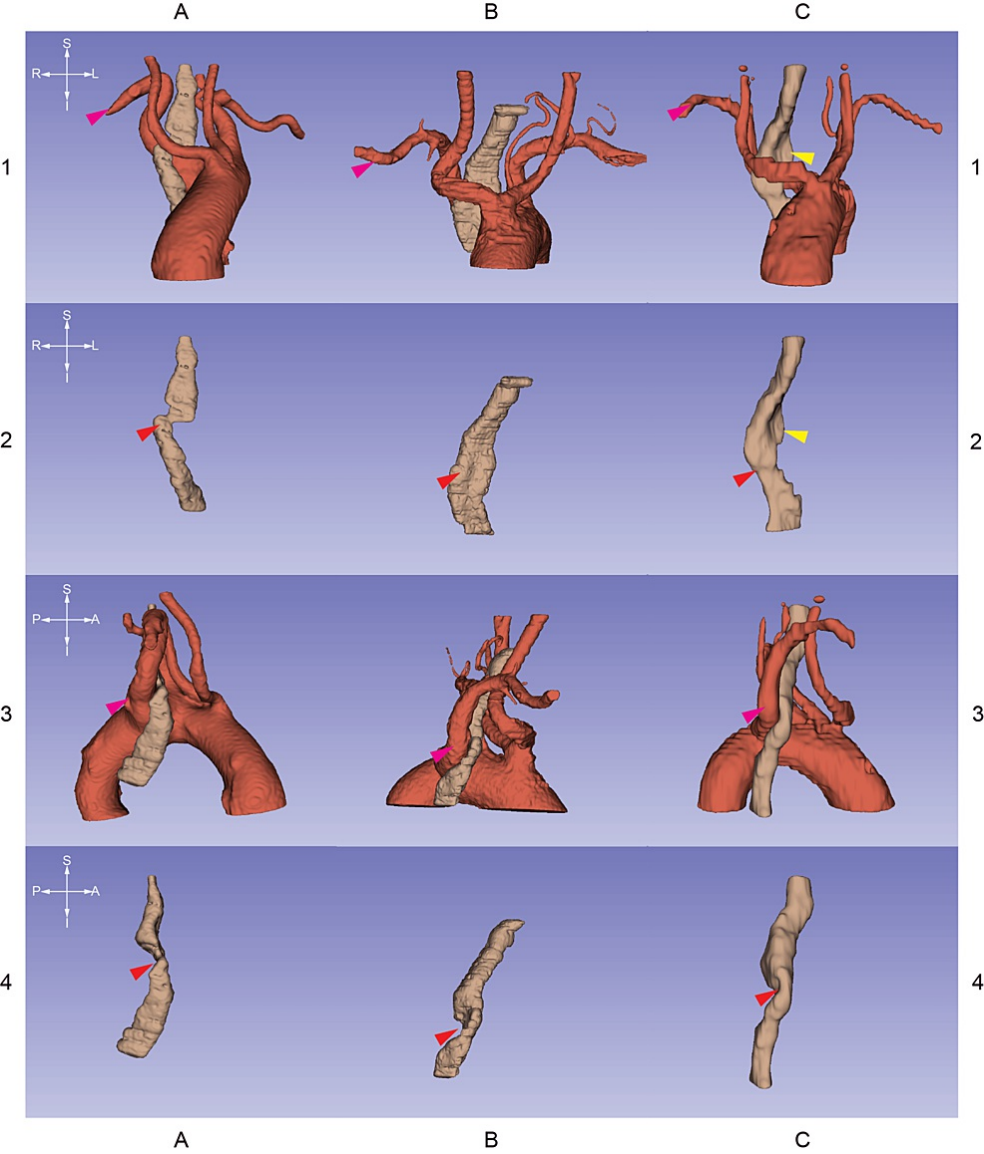
3D segmentations generated from CT stacks Columns A, B, and C are 3D segmentations derived from CT images of patients with identifiers ID036, ID230, and ID587 respectively. The crosshairs on the left are for orienting the corresponding row. The matrix demonstrates the spatial relationships between the lusorian arteries (magenta arrowheads) and the esophagi (brown segments). The aberrant right subclavian arteries form dorsal impressions on the esophagi (red arrowheads). This anterio-posterior stenosis can cause swallowing difficulties, also called "dysphagia lusoria." NB: due to the enlarged left thyroid lobe of patient ID587, there is another site of compression on the esophagus (yellow arrowheads).

Figure 2 contains CT stacks at the levels of the emerging right subclavian arteries. Figures 3-5 are 3D segmentation images generated from the CT scans. Retroesophageal emergence of the LAs (Figures 2, 4, 5) results in impressions on esophageal walls (Figure 5) which is the underlying mechanism of a possible impaired deglutition, also called dysphagia lusoria. Two of the three subjects (ID036, ID230) have Kommerell’s diverticulum (KD) (Figures 2, 3), which is a dilated, outpouching origin of the ARSA.

B1 and B2 insets in Figures 3, 4 depict the common origin of the carotid arteries (COCA) of subject ID230. This Bovine arch-like arborization of the aorta gives rise to only the two common carotid arteries due to the lack of the brachiocephalic trunk. The height of division can create a difficult anatomical situation during thyroidectomy, especially when central compartment neck dissection is also part of the surgery.

Two patients (ID230, ID587) have goiter. These conditions may require surgical treatment in the future. Care should be taken to avoid damage to the right-sided inferior laryngeal nerves which in their cases have non-recurring courses. The enlarged left thyroid lobe of patient ID587 involves the superior mediastinum (Figure 4C). It causes another site of compression on the esophagus (Figure 5C) which might worsen dysphagia.

## Discussion

As a general rule of thumb, an ARSA with a normal, left-sided aortic arch occurs in approximately 1% of individuals [9]. Therefore, the annual occurrence of a right-sided NRLN during thyroid surgery in a medium or large-volume thyroid center is almost certain, even if it is not identified during the operation [10].

Demographic analysis of the frequency of ARSA and any preoperative diagnostic attempts of this vascular anomaly, by its true nature, provides additional and substantial information on laryngeal nerve characteristics on a population and individual level. This can help the surgeon to prepare for such an anomaly in a surgical setting, and thus can also help to minimize the risk of vocal cord complications.

In this retrospective, CT-based study, three patients were identified with ARSA out of 686 imaging data. This is an estimation of a 0.437% prevalence in the Hungarian study population. This is below the widely accepted 1% ratio.

An extensive literature research providing reference of a global frequency of ARSA was also carried out including more than 100 eligible articles from the last 150 years. All of the included studies were individually reviewed and numerical data were extracted and recalculated. ARSAs with a left-sided aortic arch were taken into account exclusively. Articles that dealt with the prevalence of ARSA only in a population with congenital heart disease were also ruled out. Studies that focused on the prevalence of NRLN were only included when any imaging study had confirmed the presence of ARSA prior to surgery. Systematic review and meta-analysis of the literature were beyond the scope of the current manuscript. (Data is available upon request.) The prevalence of ARSA is between 0.00% and 5.26% in the screened literature. This wide range can be attributed to the sample sizes, investigational methods, ethnic differences, or other health conditions of the subjects. Research carried out in cardiovascular centers, for example, may present a greater prevalence since ARSA is more frequently associated with other congenital heart diseases [11]. The sensitivity of the diagnostic modality also matters because cadaver examinations and CTs can provide spatial information in detail [12]. Sample sizes are also questionable in the majority of studies. Recent meta-analyses are also available, as seen in Table 3, and they report a prevalence range between 0.56% and 0.93%; however, Natsis’ report of 8% proves that different methods in article selection can lead to outlier results [13].

**Table 3:** Summary of recent meta-analyses

Study (first author)	Year	Method	Size of screened population (n)	Number of ARSA subjects (n)	Frequency (%)
Popieluszko [14]	2017	systematic-review/meta-analysis	23882	167	0.70%
Polednak [15]	2019	systematic-review/meta-analysis	15618	88	0.56%
Recto [16]	2019	systematic-review/meta-analysis	20030	183	0.91%
Natsis [13]	2021	systematic-review/meta-analysis	4609	368	8%
Tsiouris [17]	2022	systematic-review/meta-analysis	18075	169	0.93%

It has been observed that ARSA and NRLN are more common in women, but some studies have also reported a higher incidence in men [18]. In this particular study, all cases of ARSA were found in women, which is consistent with findings from multiple centers. However, the current results are insufficient for statistical analysis. The predominance of women in NRLN cases has a significant impact on otolaryngology practice, as three times more women undergo thyroid surgery than men [19].

The LA is asymptomatic in 90% of cases. Symptoms include dysphagia, chest discomfort, and dyspnea. In rare cases, acute vascular catastrophes may occur such as rupture or arterio-esophageal fistula [20]. It is noted, that symptomatic cases are associated with KD or aneurysm of the ARSA, the rigidity of the esophagus, elongated aorta, and a bicarotid trunk [20].

The initial description and embryological definition of a KD stands for a remnant of the fourth primitive dorsal arch resulting in an outpouching origin of an ARSA [21-24]. Such a phenomenon can be visible in 60% of ARSA cases [2,21,23]. The diagnostic threshold of KD is to have a >1.5 times greater diameter at the origin than in the distal segment of the subclavian artery [25]. In the present series, two of the three subjects also had a KD (ID036, ID230), and this fits into the existing literature. This voluminous mass can facilitate the development of dysphagia-like symptoms, and angiography follow-up is recommended due to the possibility of its spatial expansion and the sequelae of a spontaneous rupture. KD is almost always present with a right-sided aortic arch (without situs inversus) and an aberrant left subclavian artery. It should be emphasized again that, due to the different embryologic patterns of this variation, a concomitant left-sided NRLN is not obvious [6,21,23].

A co-existing bicarotid trunk (also known as the common origin of the carotid arteries) can contribute to symptom development [20]. In patient no. 230 (Figure 5B), this Bovine arch-like pattern was visible. Approximately one-third of ARSA occur in combination with COCA [26]. Special care has to be taken when encountering such an anatomical variation during central neck surgery.

Thyroidectomy-related inferior laryngeal nerve damage rate is three to fivefold greater when a NRLN is present [15,27,28]. NRLN, apart from some aforementioned cases, is almost always restricted to the right side with a concomitant LA Therefore, any preoperative imaging that could detect such an anomaly of the aortic arch would be a useful tool in the head and neck surgeons’ armamentarium. For benign disease and in most cases of differentiated thyroid carcinomas ultrasound is the favorable imaging technique preoperatively. For the first time, Devèze et al. postulated the systematic use of ultrasound to detect an NRLN and ARSA based on indirect signs [29]. The lack of the Y-shaped division of the brachiocephalic trunk (Y-sign), as an indirect sign of ARSA and NRLN during the US, had a predictive value of 100%. In the past two decades, more studies have also confirmed, that the US approach of the brachiocephalic trunk has an almost 100% accuracy in predicting ARSA and NRLN, but this step hasn’t been popularized or implemented as mandatory during thyroid ultrasonography [30].

Strengths, limitations, and future perspectives

To the best of our knowledge, this is the first study that describes the prevalence of lusorian arteries among Hungarian adults. Therefore, by highlighting this vascular abnormality to head and neck surgeons, we can increase awareness about the likelihood of an NRLN.

Before making any population-wide statements, multiple methodological biases should be addressed in the future. The sample size must be increased, the gender ratio of the subjects should properly reflect the national values, and the ethnic composition of the subjects should correlate with the Hungarian society, which cannot be addressed based on the social security numbers. A machine-learning-based approach is an option to yield population-wide data in a less time-consuming manner.

Preoperative imaging can predict the occurrence of an NRLN, but it is not enough to determine the subtype and the exact course of NRLN.

## Conclusions

Based on the current findings, the frequency of an ARSA is 0.437% in Hungarian adults with a female predominance. This probability fits into the existing literature. This altered vascular patterning is in combination with a right-sided NRLN. Therefore, preoperative identification of ARSA is a reliable predictor of NRLN. Once the variation is diagnosed, its documentation can help surgeons count with an unusual course of the nerve during any operations achieved through cervical approach (e.g., thyroidectomy, carotid endarterectomy, anterior cervical spine surgery) to minimize the risk of iatrogenic vocal cord palsy.

## Appendices

It is obvious in the literature that male patients can also have a lusorian artery. The supervisor of this work also confirms that male patients had already been registered apart from this study. Sample sizes are below five in the two gender groups. Therefore, the dataset is not suitable for Pearson’s chi-square test. Fisher’s exact test didn’t serve significant results, and additionally, the lack of male patients and a low-frequency rate is not convenient for analysis.

**Table 4:** Statistical analysis of the association between sex and the prevalence of lusorian artery df: Degrees of freedom

Chi-square tests			
	ꭓ2	df	p-value
Pearson’s chi-square	3.176	1	0.075
Fisher’s exact test			0.116
